# Phosphoglycerate kinase 1 protects against ischemic damage in the gerbil hippocampus

**DOI:** 10.18632/aging.204343

**Published:** 2022-10-18

**Authors:** Kyu Ri Hahn, Hyun Jung Kwon, Yeo Sung Yoon, Dae Won Kim, In Koo Hwang

**Affiliations:** 1 Department of Anatomy and Cell Biology, College of Veterinary Medicine, Research Institute for Veterinary Science, Seoul National University, Seoul 08826, South Korea; 2 Department of Biochemistry and Molecular Biology, Research Institute of Oral Sciences, College of Dentistry, Gangneung-Wonju National University, Gangneung 25457, South Korea; 3 Department of Biomedical Sciences, Research Institute for Bioscience and Biotechnology, Hallym University, Chuncheon 24252, South Korea

**Keywords:** phosphoglycerate kinase 1, lactate, ATP, Nrf2, H_2_O_2_, neuroprotection

## Abstract

Phosphoglycerate kinase 1 (PGK1) is a metabolic enzyme that converts 1,3-diphosphoglycerate to 3-phosphoglycerate. In the current study, we synthesized a PEP-1-PGK1 fusion protein that can cross the blood-brain barrier and cell membrane, and the effects of PEP-1-PGK1 against oxidative stress were investigated HT22 cells and ischemic gerbil brain. The PEP-1-PGK1 protein and its control protein (Con-PGK1) were treated and permeability was evaluated HT22 cells. The PEP-1-PGK1 was introduced into HT22 cells depending on its concentration and incubation time and was gradually degraded over 36 h after treatment. PEP-1-PGK1, but not Con-PGK1, significantly ameliorated H_2_O_2_-induced cell damage and reactive oxygen species formation in HT22 cells. Additionally, PEP-1-PGK1, but not Con-PGK1, mitigated ischemia-induced hyperlocomotion 1 d after ischemia and 4 d after ischemia of neuronic cell death. PEP-1-PGK1 treatment significantly alleviated the raised lactate and succinate dehydrogenase activities in the early (15 min to 6 h) and late (4 and 7 d) stages of ischemia, respectively. In addition, PEP-1-PGK1 treatment ameliorated the decrease in ATP and pH levels in the late stage (2–7 d) of ischemia. Nuclear factor erythroid-2-related factor 2 (Nrf2) levels accelerated the ischemia-induced increase in the hippocampus 1 d after ischemia after PEP-1-PGK1 treatment. Neuroprotective and ameliorative effects were prominent at a low concentration (0.1 mg/kg), but not at a high concentration (1 mg/kg), of PEP-1-PGK1. Collectively, low concentrations of PEP-1-PGK1 prevented neuronal stress by increasing energy production.

## INTRODUCTION

The brain that accounts for only 2% of human body weight, consume large amounts of the total glucose in the body [1, 2]. Insufficient blood supply due to ischemia, especially glucose and oxygen transport, causes high mortality and severe complications, along with a reduced quality of life [3, 4]. Various procedure of neuronal death caused by ischemia have been proposed, along with oxidative stress and deficiency of energy consumption in the brain [5, 6]. Cerebral ischemia results in a significant step down in ATP concentration in the brain, which affects the acid–base and ionic balance to maintain neuronal homeostasis [7]. In addition, ischemia followed by reperfusion accelerates the oxidative metabolism of glucose, and production of reactive oxygen species (ROS) is enormously increased: 0.1–0.2% ROS is generated with oxygen consumption alone [8].

Most molecules of ATP are provided by mitochondrial oxidative phosphorylation; however, glycolysis is also considered a primary source of energy production [9, 10]. Under normal conditions, neurons have a low-level glycolytic state because of the low concentration of 6-phosphofructo-2-kinase/fructose-2,6-bisphosphatase-3 (PFKFB3), a key rate-limiting enzyme in glycolysis [11]. After all, ischemia followed by reperfusion significantly upregulates PFKFB3 expression in cortical neurons, which facilitates ROS production and cell death during reperfusion via aerobic glycolysis [12]. Thereafter, in pathological conditions such as ischemia, downstream glycolytic enzymes play an important role in ATP generation. Transient forebrain ischemia decreases glycolytic enzyme levels [13, 14] and we previously demonstrated that phosphoglycerate mutase 1 (PGAM1) have a neuroprotective effect on HT22 cells and ischemic gerbil brain via conversion of 3-phosphoglycerate to 2-phosphoglycerate to reduction of oxidative stress [15].

Phosphoglycerate kinase 1 (PGK1, *EC* 2.7.2.3), the upstream enzyme of PGAM1 in glycolysis, produces one molecule of ATP by catalyzing the conversion of 1,3-diphosphoglycerate and ADP to 3-phosphoglycerate and ATP [16, 17]. In several types of cancers, PGK1 expression is increased and translocated to the mitochondria. This inhibits mitochondrial pyruvate utilization to increase glycolysis production via the activation of pyruvate dehydrogenase kinase 1 [16]. Several conflicting studies have demonstrated the effects of PGK1 under various stressful conditions. PGK1 overexpression significantly reduces lactate dehydrogenase release after H_2_O_2_ treatment [18]. Moreover, the administration of terazosin, an activator of PGK1, has neuroprotective effects against focal ischemic damage [18]. In contrast, CBR-470-1, an inhibitor of PGK1, attenuates oxidative neuronal damage in 1-methyl-4-phenylpyridinium (MPP^+^) treated SH-SY5Y cells [19]. Recent studies have shown that PGK1 plays a very important role in suppressing Keap1-nuclear factor erythroid-2 related factor 2 (Nrf2) signals [20].

As a cell-penetrating peptide, PEP-1 can cross the blood-brain barrier and deliver proteins into the intracellular space. [21, 22]. We synthesized a PEP-1-PGK1 protein for effective delivery on the HT22 and gerbil hippocampal cells. One study showed that treatment with low concentrations (0.03 mg/kg) of terazosin, a PGK1 activator, protected neurons from ischemic damage, while higher concentrations (0.08 mg/kg) of terazosin had no positive effects [18]. In this study, therefore, we considered the effects of PGK1 on oxidative damage in HT22 hippocampal cells at various concentrations and against ischemic injury in the hippocampus of gerbils at both low-level (0.1 mg/kg) and high-level (1.0 mg/kg) concentrations of PEP-1-PGK1. We also elucidated the role of PGK1 in ischemia based on glycolysis, oxidative stress, and Nrf2 signaling.

## RESULTS

### Construction of PEP-1-PGK1 and its control protein (Con-PGK1) and their delivery into HT22 cells

#### Confirmation of constructed PEP-1-PGK1 and Con-PGK1 proteins

**Figure 1:**
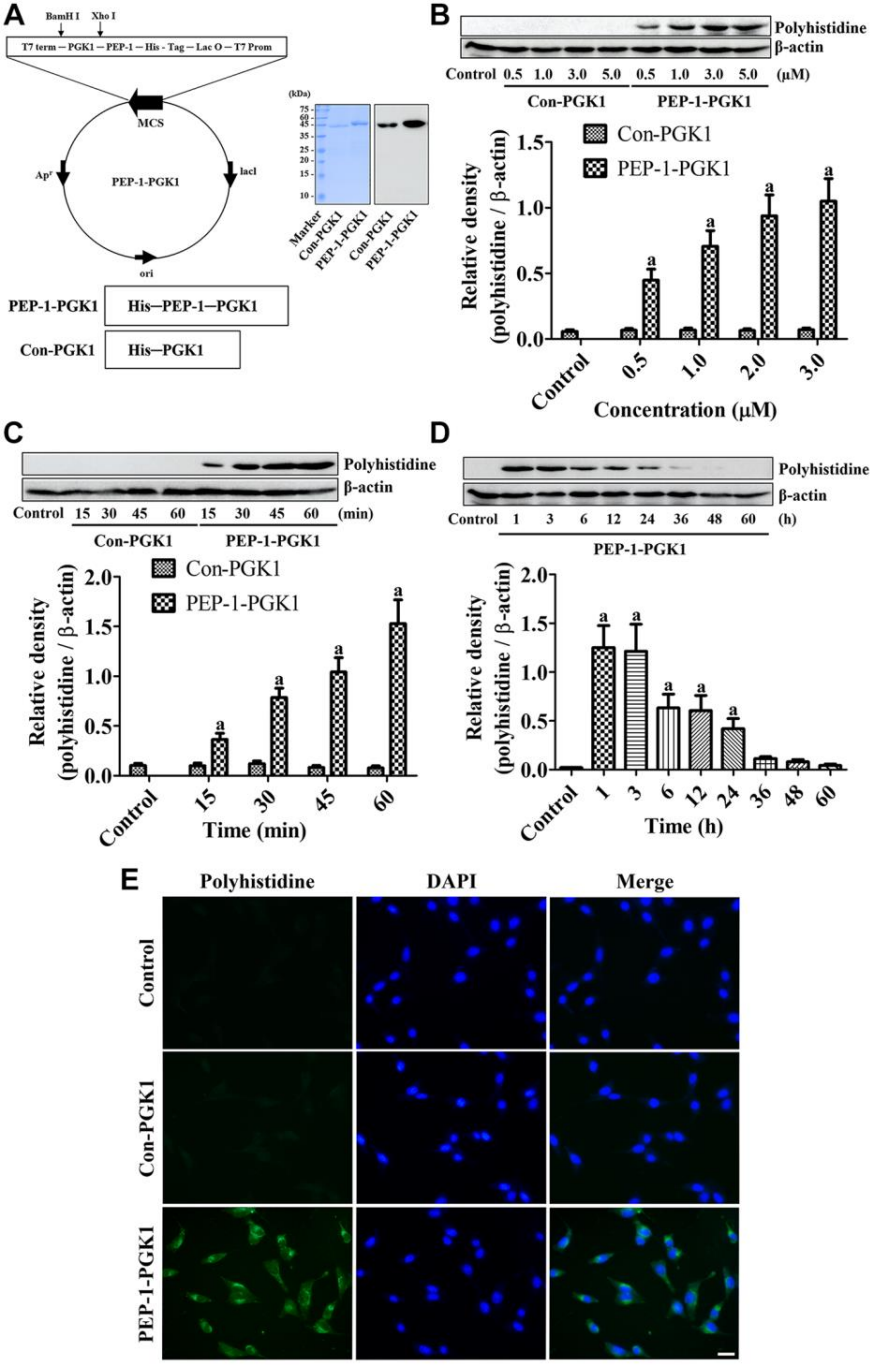
**PEP-1-PGK1 and its control protein (Con-PGK1) are synthesized, and their expressions are confirmed, in HT22 cells by visualization of a polyhistidine tag.** (**A**) PEP-1-PGK1 and Con-PGK1 are constructed, and their expressions are confirmed, in *Escherichia coli* cells. (**B**, **C**) Concentration- and time-dependent intracellular deliveries of Con-PGK1 and PEP-1-PGK1 are measured in HT22 cells after 3 μM of protein treatment and 1 h after the protein treatment, respectively. (**D**) Degradation of Con-PGK1 and PEP-1-PGK1 is assessed at various times after treatment. (**E**) Immunocytochemical staining visualizes the localization of delivered proteins in HT22 cells. Scale bar = 20 μm. (**B**–**D**) Optical densities of protein bands from western blotting are described as a value of polyhistidine/β-actin. Data are analyzed by a one-way analysis of variance, followed by a Bonferroni’s post-hoc test (^a^*p* < 0.05, significantly different from the control group). The bar graph represents the mean ± standard deviation.

The verification of the purified protein synthesized from the PEP-1-PGK1 and Con-PGK1 vectors was performed through Western blot analysis for His-tag using a polyhistidine antibody. Strong bands were detected at approximately 43 kDa and 46 kDa, respectively, by Coomassie brilliant blue staining. These proteins were confirmed to be Con-PGK1 and PEP-1-PGK1, respectively, using western blotting for polyhistidine (Figure 1A).

#### Confirmation of PEP-1-PGK1 and Con-PGK1 delivery into HT22 cells

Assessment of the concentration and time-dependent delivery of PEP-1-PGK1 and Con-PGK1 was performed by western blot analysis for polyhistidine. No significant effects of Con-PGK1 on polyhistidine levels was found comparison with the control group irrespective of concentration and time after incubation. However, a significant elevation of polyhistidine levels was observed in PEP-1-PGK1 treated group as time- and concentration dependently. Particularly polyhistidine levels were significantly higher at 0.5 μM PEP-1-PGK1 and 15 min after PEP-1-PGK1 treatment (Figure 1B, 1C).

#### Time dependent degradation of delivered PEP-1-PGK1 protein in HT22 cells

Delivered PEP-1-PGK1 and its time-dependent expression were assessed using western blotting for polyhistidine. Treatment with 3.0-μM PEP-1-PGK1 significantly elevated polyhistidine levels in HT22 cells 1 h after treatment, and polyhistidine levels dropped over time after treatment. At 36, 48, and 60 h after treatment, significant differences of polyhistidine levels were not found between the groups (Figure 1D).

#### Visualization of delivered Con-PGK1 and PEP-1-PGK1 protein in HT22 cells

Intracellular delivery of Con-PGK1 and PEP-1-PGK1 proteins was evaluated by immunochemical staining with anti-polyhistidine 1 h after 3.0-μM treatments. In the control and Con-PGK1-treated groups, immunoreaction of polyhistidine was not detectable in the HT22 cells. In contrast, in the PEP-1-PGK1 treated group, cytoplasmic polyhistidine-immunoreactive structures were observed in the HT22 cells (Figure 1E).

### Neuroprotective effect of PEP-1-PGK1 and Con-PGK1 against oxidative stress in HT22 cells

#### Determination of optimal concentration and confirmation of neuroprotection in HT22 cells

**Figure 2:**
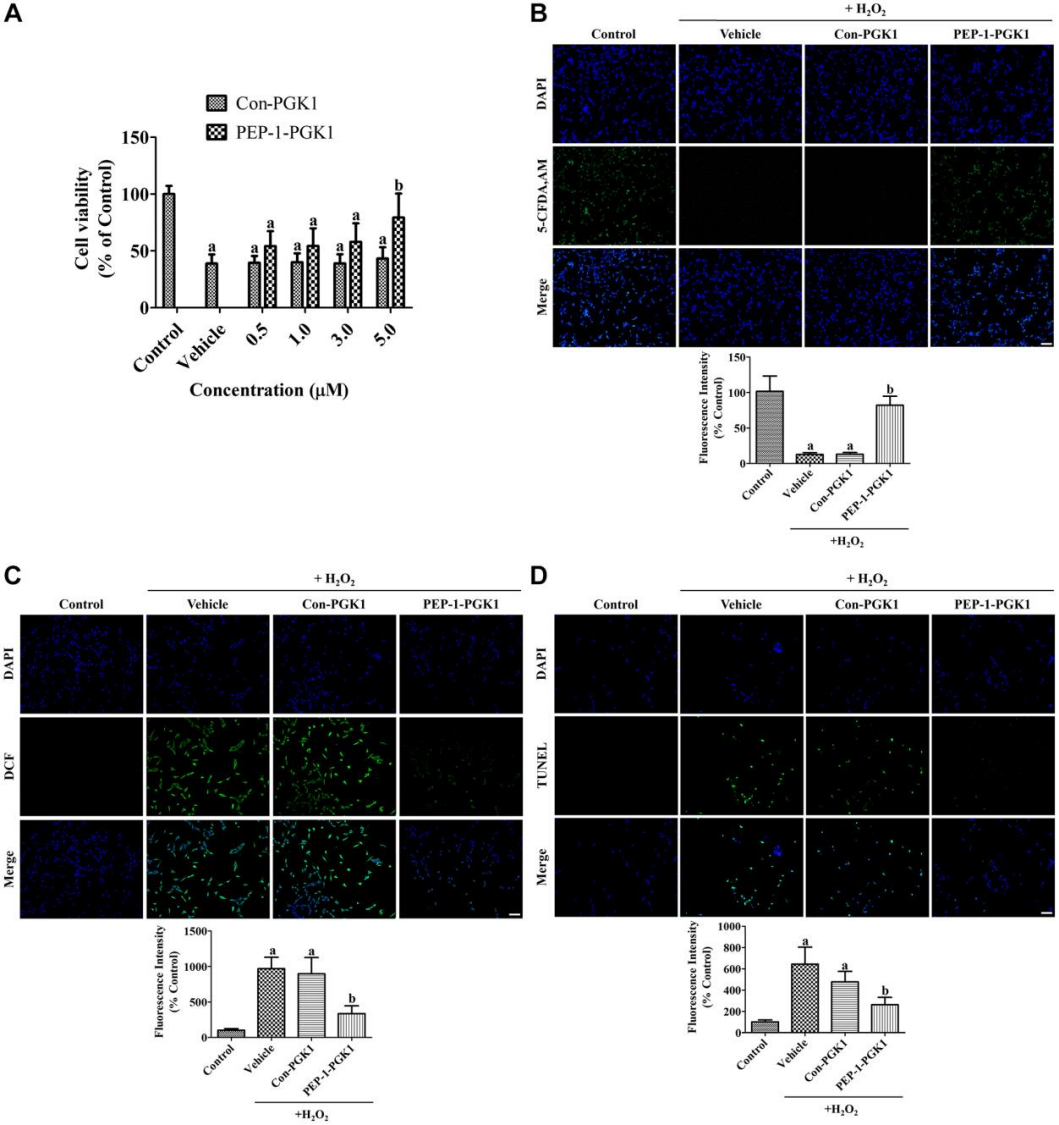
***In vitro* effects of Con-PGK1 and PEP-1-PGK1 on H_2_O_2_-induced oxidative stress in HT22 cells.** (**A**) The optimal concentration of Con-PGK1 and PEP-1-PGK1 is assessed by measurements of cell viability using a water-soluble tetrazolium salt-1 assay 1 h after H_2_O_2_, Con-PGK1, or PEP-1-PGK1 treatment. (**B**) Survived cells, (**C**) ROS formation, and (**D**) DNA fragmentation are visualized by 5-CFDA AM, DCF, and TUNEL staining, respectively, 1 h after 200 μM H_2_O_2_, 5.0 μM Con-GPK1, or 5.0 μM PEP-1-PGK1 treatment. Scale bar = 50 μm. (**B**–**D**) The intensities of 5-CFDA AM-, DCF-, and TUNEL-stained structures were spectrophotometrically measured. (**A**–**D**) Data are analyzed by a one-way analysis of variance, followed by a Bonferroni’s post-hoc test (^a^*p* < 0.05, significantly different from the control group; ^b^*p* < 0.05, significantly different from the vehicle-treated group). The bar graph represents the mean ± standard deviation.

The optimal concentrations of Con-PGK1 and PEP-1-PGK1 were determined in the treated HT22 cells. Vehicle treatment significantly reduced the viability of HT22 cells 1 h after H_2_O_2_ exposure by 38.9% compared with the control group, and the viability was increased concentration dependently with pre-incubation of PEP-1-PGK1, but not with Con-PGK1, in HT22 cells. In particular, 5-μM PEP-1-PGK1 treatment showed a significant increase of cell viability by 79.3% in the control group after 1 h of H_2_O_2_ exposure in comparison with the vehicle group (Figure 2A).

Damage on HT22 cells was visualized by staining with 5-carboxyfluorescein diacetate acetoxymethyl ester (5-CFDA AM). In the control group, 5-CFDA AM-positive cells were abundantly detected, whereas in the vehicle- and Con-PGK1-treated groups, few 5-CFDA AM-positive cells were observed 1 h after H_2_O_2_ treatment. In the PEP-1-PGK1-treated group, many 5-CFDA AM-positive cells were observed after H_2_O_2_ treatment. Significant reduction of fluorescence intensity was found in the vehicle- and Con-PGK1-treated groups by 12.7% and 13.0%, respectively, compared to that in the control group. In the PEP-1-PGK1-treated group, the reduction in fluorescence intensity induced by H_2_O_2_ treatment was significantly ameliorated to 82.0% of that in the control group (Figure 2B).

#### Effects of PEP-1-PGK1 and Con-PGK1 on H_2_O_2_-induced ROS formation and DNA fragmentation

ROS formation in HT22 cells was visualized using the fluorescent dye 2,7-dichlorofluorescein (DCF), byproduct of DCF diacetate (DCF-DA) by ROS. In the control group, DCF-positive structures were not observed, however DCF fluorescent structures were abundantly observed in HT22 cells of the vehicle- and Con-PGK1-treated groups. In the PEP-1-PGK1-treated group, few DCF positive structures were detected. Significant increases of fluorescent intensity were observed in both the vehicle- and Con-PGK1-treated groups to 969.6% and 897.8% of that in the control group, respectively. DCF fluorescence intensity in the PEP-1-PGK1-treated group significantly decreased to 336.8% of that in the control group compared with that in the vehicle- or Con-PGK1-treated groups (Figure 2C).

DNA fragmentation was assessed using terminal deoxynucleotidyl transferase dUTP nick end labeling (TUNEL) staining to detect the 3′-hydroxyl terminus of DNA breaks. In the control group, few TUNEL-positive cells were detected. However, many TUNEL-positive cells were detected in the vehicle and Con-PGK1 treatment groups. In the PEP-1-PGK1-treated group, few TUNEL-positive cells were detected in HT22 cells. Significant increase of fluorescence intensity was observed in the vehicle- and Con-PGK1-treated groups to 644.3% and 476.8% in comparison with the control group, respectively. The TUNEL fluorescence intensity of the PEP-1-PGK1-treated group was significantly lower (262.7% to the control group) than the other groups (Figure 2D).

### Neuroprotective effect of PEP-1-PGK1 and Con-PGK1 against ischemic damage in gerbils

#### Visualization of delivered Con-PGK1 and PEP-1-PGK1 protein in the gerbil hippocampus

**Figure 3:**
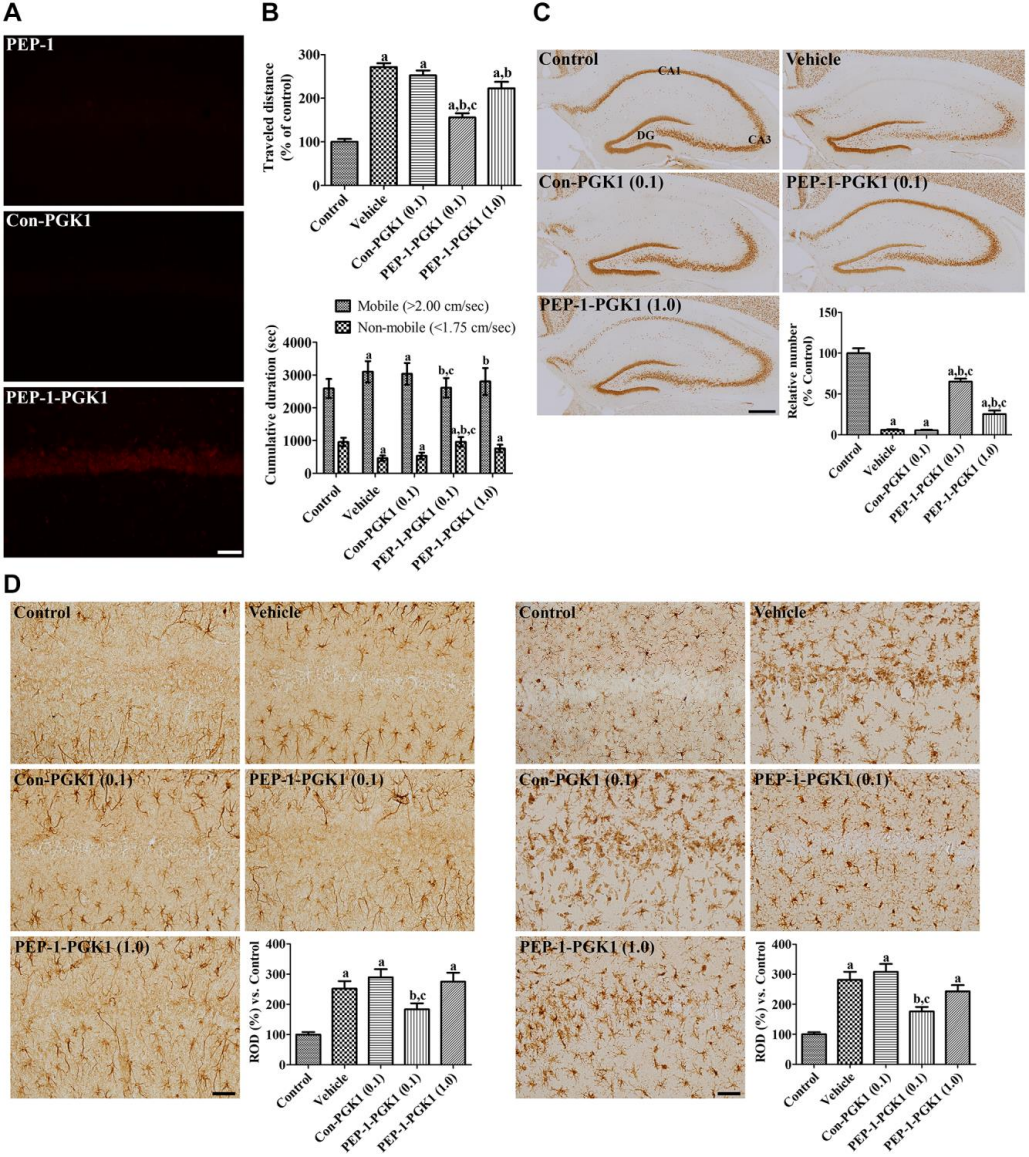
***In vivo* effects of Con-PGK1 and PEP-1-PGK1 on ischemia-induced damage in gerbils.** (**A**) Immunohistochemical staining visualizes the delivery of proteins in the gerbil hippocampus. Scale bar = 50 μm. (**B**) Motor activities of gerbils are recorded, and the travel distance and time consumed 1 d after ischemia are reanalyzed. The travel distance is expressed as a percentile value versus the control group, and the time spent in mobile and non-mobile phases is also shown. (**C**) Surviving neurons are visualized by immunohistochemical staining for NeuN in the hippocampus 4 d after ischemia. NeuN-positive neurons are counted and demonstrated as a percentile value versus the control group. Scale bar = 400 μm. (**D**) GFAP-positive astrocytes and Iba-1-positive microglia are visualized in the hippocampal CA1 region 4 d after ischemia. Scale bar = 50 μm. Relative optical densities (ROD) are expressed as a percentage of the value of GFAP and Iba-1 immunoreactivity in the hippocampal CA1 region of the control group per section, respectively. (**B**–**D**) Data are analyzed by a one-way analysis of variance, followed by a Bonferroni’s post-hoc test (*n* = 6 per group; ^a^*p* < 0.05, significantly different from the control group; ^b^*p* < 0.05, significantly different from the vehicle-treated group; ^c^*p* < 0.05, significantly different from the Con-PGK1-treated group). The bar graph represents the mean ± standard deviation.

Delivery of the PEP-1 peptide, Con-PGK1 protein, and PEP-1-PGK1 protein was evaluated by polyhistidine immunostaining at 6 h after treatment with 2 mg/kg PEP-1 or 1 mg/kg protein. In the PEP-1 peptide and Con-PGK1-treated groups, few polyhistidine-immunoreactive structures were observed in the CA1 region of the gerbil hippocampus. In contrast, in the PEP-1-PGK1-treated group, many hippocampal CA neurons were positive for polyhistidine (Figure 3A).

#### Traveled distance and time consumed in locomotion a day after ischemia

One day after ischemia, travel distance of the vehicle group was increased by 271.6% that of the control group, and the time consumed in the mobile phase of the vehicle group was longer than the control group. The 0.1 mg/kg Con-PGK1-treated group showed a similarity of the travel distance and time spent compared to those of the vehicle group. The travel distance in the 0.1 mg/kg PEP-1-PGK1-treated group was significantly shorter (155.8% of that in the control group) than that in the vehicle- or 0.1 mg/kg Con-PGK1-treated groups. Time consumption of this group was also decreased compared to the vehicle- or 0.1 mg/kg Con-PGK1-treated groups. However, in the 1.0 mg/kg PEP-1-PGK1-treated group, the travel distance as well as time spent in the mobile phase were longer than those in the 0.1 mg/kg PEP-1-PGK1-treated group (Figure 3B).

#### Surviving neurons 4 days after ischemia

In the control group, neuronal nuclei (NeuN) immunostained neurons were found in the CA1, CA3, and dentate gyrus (DG) of the hippocampus. In the vehicle-treated group, numerous NeuN-immunoreactive cells were found in the CA3 and DG regions, whereas only a few NeuN-positive cells were found in the CA1 region. The number of NeuN-positive cells was 5.9% in the control group, and 5.4% of the control group was found in the 0.1 mg/kg Con-PGK1-treated group. Many NeuN-positive cells detected in the CA1 of the 0.1 mg/kg PEP-1-PGK1-treated group. The number of NeuN-positive cells was significantly higher (65.2% of the control group) than those of the vehicle- or 0.1 mg/kg Con-PGK1-treated groups. Interestingly, in the 1.0 mg/kg PEP-1-PGK1-treated group, NeuN-immunoreactive cells in medial side of the CA1 were much lesser than in the lateral side. In this group, NeuN-positive cells were significantly increased than that of the vehicle- or Con-PGK1-treated groups, but lower than that in the 0.1 mg/kg PEP-1-PGK1-treated group (Figure 3C).

#### Reactive gliosis 4 days after ischemia

In the control group, glial fibrillary acidic protein (GFAP)-positive astrocytes and ionized calcium-binding adapter molecule 1 (Iba-1)-positive microglia were mainly detected in the strata radiatum and oriens, which had a small cytoplasm with thin processes. In the vehicle- and Con-PGK1-treated groups, GFAP-positive astrocytes and Iba-1-positive microglia had hypertrophied cytoplasm with thickened processes. In addition, Iba-1-positive microglia aggregated in the stratum pyramidale, which had a rounded cytoplasm with rarely developed processes. In the 0.1 mg/kg PEP-1-PGK1-treated group, GFAP-positive astrocytes were mixed with thin and hypertrophied cytoplasm. In this group, Iba-1-positive microglia had hypertrophied cytoplasm, but few microglia were observed in the stratum pyramidale. In the 1.0 mg/kg PEP-1-PGK1-treated group, hypertrophied astrocytes and microglia were found in the CA1 region, and microglia aggregated in the stratum pyramidale on the medial side of the CA1 region. Significantly increases of GFAP and Iba-1 immunoreactivities were found in the vehicle-treated group compared to those of the control group, to 251.8% and 281.3% respectively. GFAP and Iba-1 Immunoreactivity of the Con-PGK1 group was not significantly different to the vehicle group, but it significantly lower than the 0.1 mg/kg PEP-1-PGK1 group. GFAP and Iba-1 immunoreactivity of the 1.0 mg/kg PEP-1-PGK1 group were not showed significant differences in comparison with the vehicle-treated group (Figure 3D).

### Mechanisms of PEP-1-PGK1 and Con-PGK1 against ischemic damage in gerbils

#### Succinate dehydrogenase activity, pH, ATP, lactate, and malondialdehyde levels

In the hippocampus of control group, no significant changes were observed in succinate dehydrogenase (SDH) activity, pH, ATP, lactate, or malondialdehyde (MDA) levels. In the vehicle- and Con-PGK1-treated groups, SDH activity gradually significantly increased at 4 and 7 d after ischemia. In the 0.1 mg/kg PEP-1-PGK1-treated group, SDH activity was significantly lower 4 and 7 d after ischemia compared to that in the vehicle-treated group. However, in the 1.0 mg/kg PEP-1-PGK1-treated group, SDH activity was not significantly differ to that of the vehicle group on ischemic duration.

In the vehicle- and Con-PGK1-treated groups, pH levels gradually decreased and it was significantly lower at 6 h and at 2, 4, and 7 d after ischemia in comparison with those of corresponding control group. In the 0.1 mg/kg PEP-1-PGK1-treated group, pH levels of 6 h, 4 d, and 7 d after ischemia were significantly higher than those in the time-matched vehicle-treated group. However, pH levels of the 1.0 mg/kg PEP-1-PGK1-treated group were significantly increased only at 7 d in comparison with those of the vehicle-treated group.

ATP contents of the vehicle- and Con-PGK1-treated groups were decreased after ischemia and was significantly lower than control group at 2, 4, and 7 d after ischemia. In the 0.1 mg/kg PEP-1-PGK1-treated group, ATP content of 0.1 mg/kg PEP-1-PGK1 group was significantly higher than the vehicle-treated group at 6 h, 2 d, 4 d, and 7 d after ischemia. However, in the 1.0 mg/kg PEP-1-GPK1-treated group, ATP level were not significantly differed to vehicle-treated group at corresponding time point.

In the vehicle- and Con-PGK1-treated groups, lactate levels were significantly higher at 15 min and 6 h after ischemia. After that, lactate levels reduced over time in comparison with those in the time-matched control group. In the 0.1 and 1.0 mg/kg PEP-1-PGK1-treated groups, lactate levels were significantly lower 15 min and 6 h after ischemia than those in the time-matched vehicle-treated group.

**Figure 4:**
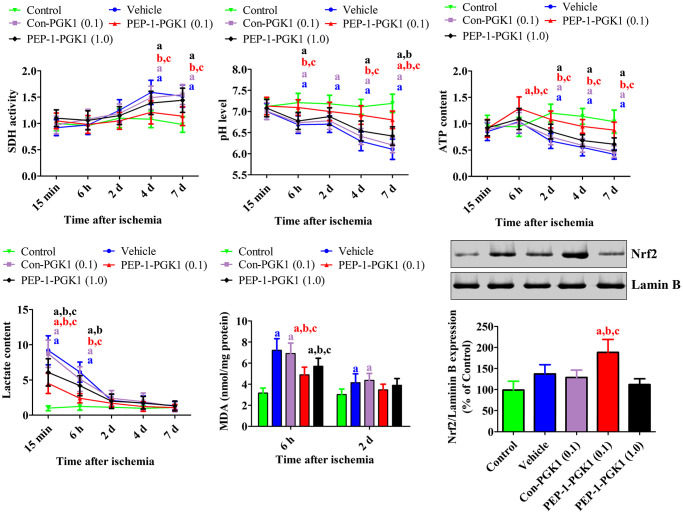
***In vivo* mechanisms of Con-PGK1 and PEP-1-PGK1 on ischemia-induced damage in gerbils.** Succinate dehydrogenase activity, pH, ATP, lactate, and malondialdehyde levels were assessed in the hippocampus of gerbils at various times after ischemia. The nuclear fraction of nuclear factor erythroid-2-related factor 2 was measured in the hippocampus using western blotting 1 d after ischemia. Data are analyzed by a one-way analysis of variance, followed by a Bonferroni’s post-hoc test (*n* = 6 per group; ^a^*p* < 0.05, significantly different from the control group; ^b^*p* < 0.05, significantly different from the vehicle-treated group; ^c^*p* < 0.05, significantly different from the Con-PGK1-treated group). The bar graph represents the mean ± standard deviation.

In the vehicle- and Con-PGK1-treated groups, MDA levels were significantly higher 6 h and 2 d, respectively, after ischemia than those in the control group. At 6 h after ischemia, MDA levels of the 0.1 and 1.0 mg/kg PEP-1-PGK1 groups were significantly lower than the control group (Figure 4).

#### Nrf2 levels

Nrf2 levels were significantly altered in the nuclear fraction of the gerbil hippocampus 1 d after ischemia. In the vehicle- and Con-PGK1-treated groups, Nrf2 levels in the nuclear fraction were 137.3% and 128.6% higher, respectively, than those in the control group. Nrf2 levels of the 0.1 mg/kg PEP-1-PGK1-treated group further increased in the nuclear fraction to 188.3% of that of the control group compared with those of vehicle-treated group. However, Nrf2 levels of 0.1 mg/kg PEP-1-PGK1 groups were lower than the vehicle group (Figure 4).

## DISCUSSION

PGK1, a glycolytic enzyme, catalyzes the alteration of 3-phosphoglycerate and ATP from 1,3-diphosphoglycerate and ADP [16, 17]. In previous studies, we observed that PGAM1, a glycolytic enzyme, ameliorated neuronal damage from oxidative stress [15, 23]. We synthesized PEP-1-PGK1, a cell-permeable protein, and its control protein, Con-PGK1, into an *E. coli* strain. Treatment with PEP-1-PGK1, but not Con-PGK1, transduced intracellularly time- and concentration dependently in HT22 cells, and PEP-1-PGK1 was stably expressed on HT22 cells 15 min after treatment. In addition, PEP-1-PGK1 pre-incubation significantly reduced H_2_O_2_-induced cell death, DNA fragmentation, and ROS production in HT22 cells. This result is resembled with those of previous studies showing that PGK1 mitigates amyloid-induced apoptosis in human SH-SY5Y cells [24, 25]. Moreover, treatment with a PGK1 activator decreased cell death induced by H_2_O_2_. Blocking PGK1 shRNA also abolished neuroprotective effects [18]. In contrast, silencing or knockout of PGK1 protected osteoblasts from dexamethasone-induced oxidative damage by activating Nrf2 signaling [26]. Furthermore, inhibition of PGK1 resulted in decreased cellular toxicity induced by MPP^+^ treatment through the activation of the Keap1–Nrf2 cascade [19].

To extend our observations PEP-1-PGK1 was applied to ischemic gerbil model. We found that PEP-1-PGK1, but not PEP-1 peptide or Con-PGK1, was delivered to the gerbil hippocampus, and 0.1 mg/kg PEP-1-PGK1 treatment effectively improved hyperlocomotion and hippocampal neuronal death induced by ischemia in the gerbils, while a higher (1.0 mg/kg) concentration of PEP-1-PGK1 had no protective effects against ischemia. This result is corresponded well with a previous study that demonstrated neuronal protection against ischemic damage in the presence of a low concentration (0.03 mg/kg) of terazosin, a PGK1 activator. In contrast, positive effects were not found at a higher concentration (0.08 mg/kg) of PGK1 activator treatment [18]. This neuroprotective effect was also blocked by PGK1 shRNA via the activation of caspase-3 and cleavage of PARP-1 [18]. However, inhibition of PGK1 activates the Keap1–Nrf2 cascade [20, 26] and protects osteoblasts from dexamethasone-induced oxidative damage [26]. Furthermore, inhibition of PGK1 by CBR-470-1 mitigates oxidative injury and neuronal damage in SH-SY5Y cells after MPP+ treatment [19].

To clarify neuroprotective effects of PEP-1-PGK1 in ischemic impairments, pH, ATP, and lactate levels for energy metabolism, including MDA and nuclear Nrf2 levels for oxidative/antioxidant responses in the hippocampus of gerbils were investigated. In this study, we recognized that transient forebrain ischemia resulted in acidosis in the hippocampus with increased lactate levels and decreased ATP levels, which is same with the results of earlier studies showing that pH and ATP levels significantly decreased after ischemia [27–29]. We also observed significant increases in MDA and nuclear Nrf2 levels in the hippocampus following ischemia. MDA levels were notably elevated and maintained from 6 h to 4 d after ischemia [30]. Moreover, lipid peroxidation inhibitors attenuated neuronal damage induced by ischemia in gerbils [31]. In contrast, Nrf2 expression was higher 24 h after ischemia in the hippocampus of gerbils [32], probably to compensate for the ROS-induced hippocampal neuronal damage due to a significant increase in antioxidant enzymes 1 in CA1 region of the hippocampus 1 d after ischemia [33, 34]. In current study, we noticed that 0.1 mg/kg PEP-1-PGK1 treatment remarkably reduced the decreases of ischemia-induced pH and ATP levels in the hippocampus, and it also increased lactate and MDA levels after ischemia. However, higher concentrations of PEP-1-PGK1 (1.0 mg/kg) did not show these prominent effects. These results show that low concentrations of PEP-1-PGK1 significantly mitigate ischemia-induced oxidative impairment by decreasing lipid peroxidation and increasing hippocampal ATP levels. This result is continued with a preceding study that terazosin showed increase of ATP levels in the brain, which ameliorates the progression of Parkinson’s disease [35]. We also noticed that PEP-1-PGK1 treatment significantly elevated hippocampal nuclear Nrf2 levels, suggesting that PEP-1-PGK1 treatment facilitates the translocation of Nrf2 to the nucleus and increases antioxidant response elements with small Maf transcription factors, which promote the generation of antioxidant enzymes and anti-inflammatory cytokines [36–38]. In addition, ATP stimulates microglial activation, and activated Nrf2 plays a critical role in microglial phenotypic changes from a pro- to an anti-inflammatory [39, 40]. In this study, we demonstrate an increase in ATP and nuclear Nrf2, suggesting that treatment with a low concentration of PEP-1-PGK1 facilitates anti-inflammatory and antioxidant responses in the hippocampus after ischemia. However, at higher concentrations, nuclear Nrf2 translocation was significantly decreased in hippocampal region, suggesting that Nrf2 expression in the nucleus may explain why PEP-1-PGK1 has conflicting point against ischemic injury in gerbils.

In conclusion, we observed that PEP-1-PGK1 reduced oxidative stress in HT22 cells and also reduced ischemic injury in gerbils. The neuroprotective effects of PGK1 are closely associated with the maintenance of energy metabolism and antioxidant properties in the hippocampus. These results suggest that glycolytic activity could present a strategy for the development of therapeutic agents against oxidative and ischemic damage.

## MATERIALS AND METHODS

### Synthesis of PEP-1-PGK1 and Con-PGK1 and their delivery into HT22 cells

#### Synthesis of PEP-1-PGK1 and control PGK1 proteins

PEP-1 peptide (KETWWETWWTEWSQPKKKRKVC) was purchased from Peptron Co. (Daejeon, South Korea), and PEP-1-PGK1 was synthesized by cloning human PGK1 cDNA in a TA vector and subcloning into a PEP-1 expression vector containing a polyhistidine tag, as noted previously [41]. Control PGK1 (Con-PGK1) was synthesized without the PEP-1 peptide. PEP-1-PGK1 or Con-PGK1 plasmids were cloned into *Escherichia coli* strain BL21 (DE3), and protein overexpression was induced by incubation with 0.5 mM isopropyl-β-D-thiogalactoside. After cell harvest and lysis, PEP-1-PGK1 and Con-PGK1 were purified and desalted using a Ni^2+^-nitrilotriacetic acid Sepharose column (Qiagen, Inc., Chatsworth, CA, USA) and PD-10 desalting column chromatography (GE Healthcare, Piscataway, NJ, USA), respectively.

Finally, the purified PEP-1-PGK1 and Con-PGK1 proteins were confirmed by western blot analysis for the His-tag, as described previously [41]. Protein bands were visualized using chemiluminescent reagents according to the manufacturer’s recommendations (Amersham, Franklin Lakes, NJ, USA).

#### Delivery of PEP-1-PGK1 and Con-PGK1 proteins into HT22 cells

HT22 hippocampal cells, purchased from ATCC (Manassas, VA, USA), were cultivated in Dulbecco’s modified Eagle’s medium, as described previously [41]. Various concentrations (0.5–5.0 μM) of PEP-1-PGK1 and Con-PGK1 proteins were added to HT22 cells. The cells were harvested 1 h after protein treatment. Additionally, 1.0 μM of proteins were incubated in HT22 cells, and the cells were harvested 15–60 min after treatment. Protein delivery was confirmed by western blot analysis for polyhistidine via the detection of the His-tag, as described previously [15, 41].

#### Degradation of delivered PEP-1-PGK1 protein in HT22 cells

PEP-1-PGK1 protein (1.0 μM) was added to HT22 cells. The cells were harvested 1–60 h after treatment to detect degradation of the delivered PEP-1-PGK1 protein. The PEP-1-PGK1 protein was assessed using western blot analysis for the His-tag, as described previously [15, 41].

#### Morphological findings of delivered PEP-1-PGK1 and Con-PGK1 proteins in HT22 cells

To confirm the intracellular delivery of PEP-1-PGK1 and Con-PGK1 in HT22 cells, 1.0 μM PEP-1-PGK1 or Con-PGK1 proteins were incubated for 1 h in HT22 cells grown on coverslips. The cells were fixed with 4% paraformaldehyde in 0.1 M phosphate buffer (pH 7.4) for 5 min at 25°C. PEP-1-PGK1 and Con-PGK1 were visualized via immunohistochemical staining for polyhistidine by counterstaining with 1 μg/mL 4′,6-diamidino-2-phenylindole (Roche Applied Science, Mannheim, Germany), as described previously [15, 41].

### Effect of PEP-1-PGK1 and Con-PGK1 on cellular damage against oxidative stress in HT22 cells

#### Determination of optimal concentration to prevent neuronal death in HT22 cells

The optimal concentrations of PEP-1-PGK1 and Con-PGK1 for neuroprotective effects against oxidative stress were determined using a water-soluble tetrazolium salt-1 assay. Oxidative stress was induced by treatment with 200 μM H_2_O_2_ and PEP-1-PGK1, and Con-PGK1 (0.5–5.0 μM) was treated simultaneously with H_2_O_2_. One hour after H_2_O_2_, PEP-1-PGK1, or Con-PGK1 treatment, cells were harvested and cell viability was assessed by measuring formazan formation based on the optical density at 450 nm using an enzyme-linked immunosorbent assay (ELISA) microplate reader (Labsystems Multiskan MCC/340, Helsinki, Finland), as described previously [15, 41].

#### Cell viability, DNA fragmentation, and ROS levels

Cell viability, DNA fragmentation, and ROS formation were visualized via 5-CFDA AM (Invitrogen), TUNEL, and DCF-DA staining, as described previously [15, 41]. HT22 cells were incubated with 200 μM H_2_O_2_, 5.0 μM PEP-1-PGK1, or Con-PGK1 for 1 h and then treated for 20 min at 37°C with 1 μM 5-CFDA AM or with 10 μM DCF-DA. For TUNEL staining, cells were fixed with 4% paraformaldehyde 1 h after H_2_O_2_ treatment and stained according to the manufacturer’s guidelines. Stained structures in the HT22 cells were observed using a fluorescence microscope (Nikon Eclipse 80i, Tokyo, Japan). Fluorescence intensities were measured using a Fluoroskan ELISA plate reader (LabSystems Oy, Helsinki, Finland).

#### Effect of PEP-1-PGK1 and Con-PGK1 on ischemic damage in gerbils

#### Experimental animals and confirmation of intracellular delivery of proteins

Mongolian gerbils (male, 6 weeks old) were obtained from Japan SLC Inc. (Shizuoka, Japan). The animals were acclimatized for 2 weeks. Animal study procedures were approved by the Institutional Animal Care and Use Committee of Seoul National University (SNU-200313-2-4). PEP-1-PGK1 (0.1 or 1.0 mg/kg) and Con-PGK1 (0.1 mg/kg) proteins were intraperitoneally administered to gerbils. The gerbils were sacrificed 6 h after 2 mg/kg peptide, 0.1 mg/kg PEP-1-PGK1, and 0.1 mg/kg Con-PGK1 treatment to visualize the intracellular delivery of proteins based on immunohistochemical staining for polyhistidine.

#### Induction of transient forebrain ischemia and treatment of PEP-1-PGK1 or Con-PGK1

Animals were anesthetized with 2.5% isoflurane (Baxter, Deerfield, IL, USA) mixed with nitrous oxide and oxygen. A ventral neck incision was made, and both common carotid arteries were occluded with aneurysm clips for 5 min under body temperature regulation (37.0°C ± 0.3°C) using a thermostatic blanket connected to a rectal probe. Vascular interruption and reperfusion were confirmed using a surgical stereoscope and an ophthalmoscope (HEINE K180^®^, Heine Optotechnik, Herrsching, Germany). The sham operation was performed using the same surgical procedures, but without occlusion of the common carotid arteries, and sham-operated animals were considered to be the control group. Immediately after reperfusion, animals received intraperitoneal injections of PEP-1 peptide (2 mg/kg), PEP-1-PGK1 protein (0.1 or 1.0 mg/kg), or Con-PGK1 protein (1.0 mg/kg).

#### Spontaneous motor activity

Locomotor activities (travel distance and time in immobile/mobile phases) were tracked in gerbils 1 d after the onset of ischemia, because that is when they were significantly increased. Animal activity was traced using a digital camera (Basler, Ahrensburg, Germany) for 60 min. The time spent in the immobile/mobile phases and the distance traveled were analyzed using XT14 software (Ethovision, Wageningen, Netherlands), as described previously [15, 41].

#### Neuronal damage and reactive gliosis

Immunohistochemical staining for NeuN, GFAP, and Iba-1 was performed to visualize surviving neurons, astrocytes, and microglia in the hippocampus 4 d after ischemia. The animals were re-anesthetized with isoflurane and perfused transcardially with physiological saline and 4% paraformaldehyde via the left ventricle. The part of the brain located on the gerbil atlas 1.4–2.0 mm caudal to the bregma [42] was sectioned at a thickness of 30-μm using a sliding microtome (HM430, Thermo Scientific, Waltham, MA, USA). Five sections, at 90-μm intervals from each other, were incubated with mouse anti-NeuN antibody (1:1000; EMD Millipore, Temecula, CA, USA), rabbit anti-GFAP antibody (1:1000; EMD Millipore), and rabbit anti-Iba-1 antibody (1:500; Wako, Osaka, Japan) for 48 h at 4°C. After sequential treatment with biotinylated goat anti-mouse IgG or anti-rabbit IgG and streptavidin–peroxidase complex (1:200; Vector, Burlingame, CA, USA) for 2 h at 25°C, immunoreactions were developed using 3,3-diaminobenzidine tetrachloride (Sigma).

### Mechanisms of PEP-1-PGK1 and Con-PGK1 on ischemic damage in gerbils

#### Measurements of pH, ATP, lactate, and lipid peroxidation levels

Energy production, acidosis-related markers, and lipid peroxidation markers were validated to elucidate the neuroprotective effects of PEP-1-PGK1 against ischemic damage in the hippocampus of gerbils. Animals were sacrificed with isoflurane, and their hippocampi were dissected and homogenized in the assay buffer. Intracellular pH, ATP, lactate, and lipid peroxidation levels were measured using commercially available assay kits (a fluorometric intracellular pH assay kit [Merck, Darmstadt, Germany], ATP determination kit [Molecular Probes], lactate colorimetric assay kit [Abcam, Cambridge, UK], and MDA assay kit [Abcam], respectively), according to each manufacturer’s protocol.

#### Western blot analysis for Nrf2

Nrf2 protein levels were assessed by western blot analysis to observe the effects of PEP-1-PGK1 on Nrf2-mediated antioxidant cascades, as stated previously [32]. The animals were sacrificed with isoflurane 1 d after ischemia. The hippocampi were dissected, homogenized, and loaded onto a sodium dodecyl sulfate–polyacrylamide gel for electrophoresis. Thereafter, the gel was transferred onto a nitrocellulose membrane (Pall Crop, East Hills, NY, USA), which was incubated with rabbit anti-Nrf2 (1:1000, Abcam) and laminin B (Santa Cruz Biotechnology, Santa Cruz, CA, USA). Protein bands were visualized using a chemiluminescence solution (GE Healthcare, Buckinghamshire, UK), and Nrf2 protein bands were normalized to those in laminin B.

### Data quantification and analysis

#### Quantification of immunohistochemical data

In the five sections at 90-μm intervals, the number of NeuN-immunoreactive neurons was counted at the mid-point of the hippocampal CA1 region. GFAP and Iba-1 immunoreactivity was quantified as optical density and pixel numbers using ImageJ software version 1.80 (National Institutes of Health, Bethesda, MD, USA), as described previously [15, 32]. Data are presented as percentage values compared with the control group values (set at 100%).

### Statistical analysis

Data are conferred as means ± standard deviations. Differences in means were compared and statistically analyzed using one-way or two-way analyses of variance, followed by Bonferroni’s post-hoc tests, using GraphPad Prism 5.01 software (GraphPad Software, Inc., La Jolla, CA, USA).
